# The Impact of Administering Human Plasma Protein Fraction 5% (PPF5%) in Major Liver Resection Surgeries

**DOI:** 10.7759/cureus.77824

**Published:** 2025-01-22

**Authors:** Yasser Hammad, Hazim Kassas, Tarek Tageldin, Muhammad Jaffar Khan, Muhammad Firas Alhammad, Walid Elmoghazy, Ahmed Elaffandi, Mohamed Yahia Koura, El Sayed Mohamed El Karta, Nabil A Shallik

**Affiliations:** 1 Anesthesiology and Critical Care, Hamad Medical Corporation, Doha, QAT; 2 Clinical Anesthesiology, Weill Cornell Medicine-Qatar, Doha, QAT; 3 Hepatobiliary and Liver Transplant Surgery, Hamad Medical Corporation, Doha, QAT; 4 Surgery, Hamad Medical Corporation, Doha, QAT; 5 Surgical Oncology, National Cancer Institute, Cairo University, Giza, EGY; 6 Anesthesiology and Intensive Care Unit, Damietta Faculty of Medicine, Al-Azhar University, Damietta, EGY; 7 Clinical Anesthesiology, Qatar University, Doha, QAT; 8 Clinical Anesthesiology and Surgical Intensive Care Unit, Faculty of Medicine, Tanta University, Tanta, EGY

**Keywords:** acid-base balance, crystalloids, electrolyte, human plasma protein fraction, liver resection, ringer lactate solution, tissue perfusion

## Abstract

Introduction

Albumin substantially influences the acid-base equilibrium within the human body and the regulation of acid-base homeostasis. The precise role of albumin remains a subject of debate. Human plasma protein fraction 5% (PPF5%) (Octapharma Pharmazeutika Produktionsgesellschaft m.b.H., Vienna, Austria) contains selected plasma with approximately 88% normal human albumin. We hypothesize that the use of PPF5% in patients undergoing liver resection surgery will enhance tissue perfusion and augment the buffering capacity of blood.

Methods

A prospective, randomized controlled study spanning 18 months was conducted at Hamad General Hospital, Qatar, involving 48 patients scheduled for liver resection surgeries. Patients were allocated into two groups: group A received an intravenous infusion of PPF5%, while group R received an intravenous infusion of crystalloid (lactated Ringer's solution). Acid-base electrolyte fluctuations were evaluated on four occasions at different time intervals.

Results

There were significant alterations within each group at different time points. Group A exhibited a substantial variance in strong ion difference (SID) values compared to group R at the time after liver resection (TAR) and at the time of end of surgery (TE). Group A demonstrated significantly enhanced tissue perfusion at TAR and TE relative to group R. There was a noteworthy decrease in hemoglobin and hematocrit levels in group A compared to group R at TE due to the hemodilution effect of the PPF5%.

Conclusions

The use of PPF5% in significant quantities for rehydration during liver resection procedures appears safe, exhibiting no equivalent alterations in acid-base balance, electrolyte levels, and coagulation when compared to lactated Ringer's solution. Moreover, it demonstrates improved tissue perfusion alongside increased hemodilution.

## Introduction

Human plasma protein fraction 5% (PPF5%) and crystalloids serve as plasma expanders and are utilized to replenish blood and fluid loss during liver resection procedures. Albumin, containing histidine residues with an acid dissociation constant, serves as a significant buffer donor of positive charges during alkalosis and negative charges during acidosis [1,2]. Conversely, some researchers contend that albumin does not function as a buffer and may diminish plasma buffer strength [3]. In accordance with Stewart's model, two primary factors influence the metabolic component of blood: the strong ion difference (SID), primarily comprising sodium, chloride, and lactate, and the total weak acid concentration, primarily involving albumin [4]. Acidosis arises from a narrow SID, such as low sodium or high chloride levels, and the accumulation of lactic acids. Additionally, the accumulation of weak acids independently contributes to pH changes [5].

Furthermore, fluid infusion induces dilution acidosis owing to the linear alteration in SID. Concurrently, it dilutes weak acids, primarily albumin, resulting in an alkalotic effect that mitigates acidosis triggered by diminished SID. Solutions containing albumin exhibit an acidotic effect due to its anionic properties. Nevertheless, the in vivo influence on SID (widening effect) mitigates pH alterations [6,7].

Standard base excess (SBE) serves as a metric for quantifying the fluid impact on acid-base equilibrium [4]. It denotes the quantity of acid or base required to restore one liter of extracellular fluid to a normal pH (7.40) under standardized conditions (37°C and arterial carbon dioxide tension (PaCO2) of 40 mmHg). Its range spans from -3 to +3 mEq/L [4]. In the absence of chronic respiratory alterations, SBE is determined by SID, variations in albumin concentration, and the accumulation of other anions.

Any alterations in acid-base balance and electrolyte levels hold clinical significance, particularly in the management of patients susceptible to fluctuations in blood chemistry, such as those with liver disease undergoing surgical interventions.

These fluctuations impact hemodynamic stability, coagulation, tissue perfusion, and various other physiological processes. Research examining the acid-base and electrolyte alterations linked to the administration of chloride-rich crystalloids during significant non-hepatic surgeries has demonstrated the occurrence of dilution acidosis through electrolyte redistribution, potentially inducing hyperchloremic acidosis, which could lead to acute kidney injury [8,9].

Liver surgery is further linked with hypovolemia and diminished vascular tone. Moreover, albumin serves as a crucial factor in determining plasma osmotic pressure, alongside its multifaceted roles as a carrier for various compounds, a scavenger, an antioxidant, and an exertor of anti-inflammatory effects. Additionally, it acts as a buffer molecule contributing to pH homeostasis [1,2].

Hypoalbuminemia consequently compromises the perfusion and buffering capacity of the circulating blood. Nevertheless, the influence of albumin-rich PPF5% on acid-base and electrolyte equilibrium during liver surgery remains insufficiently explored in the existing literature.

In this current investigation, our hypothesis posits that employing albumin-rich PPF5% either as the sole solution or in conjunction with crystalloids in patients undergoing liver resection surgery will enhance tissue perfusion and augment the blood's buffering capacity.

The primary endpoint of the study encompasses alterations in blood acid-base status and electrolytes, assessed through Stewart's quantitative acid-base chemistry, specifically focusing on $\text{Strong ion difference (SID)}=\text{Sodium (Na+)}-\text{Chloride (Cl-)}-\text{(Lactate})$, as well as pH and base deficit measurements. Secondary endpoints include variations in perfusion markers such as urine output, lactic acid levels, and the calculated transcutaneous carbon dioxide tension (PtcCO2)/inspired oxygen concentration (FiO2) ratio, alongside the perfusion index. Additionally, parameters such as hemoglobin and hematocrit concentrations, intravascular albumin mass (IAM), changes in plasma volume (PV), blood loss, and clotting index are also evaluated.

## Materials and methods

A total of 48 patients, aged between 18 and 70 years, scheduled for liver resection surgeries at Hamad General Hospital, Qatar, were enrolled in a prospective, randomized controlled study conducted over a span of 18 months. The study received approval from the Institutional Review Board of Hamad Medical Corporation (approval number: HMC-IRB-MRC-01-18-256) on November 15, 2022, and written informed consent was obtained from all participants. Subjects were electronically randomized into two groups, namely, group A and group R. Patients with cardiac disease and liver dysfunction were excluded from the study. Each patient underwent a comprehensive medical and surgical history review, as well as a thorough clinical examination. Routine blood tests, including complete blood count, kidney function tests (serum urea, creatinine, sodium, and potassium), liver function tests (alanine transaminase, aspartate transaminase, prothrombin time and concentration, international normalized ratio, and serum albumin), random blood sugar assessment, and serum electrolyte analysis (calcium, chloride, bicarbonate, and lactate), were performed for all participants.

Twenty-five out of the 48 patients received an intraoperative intravenous infusion of PPF5% (designated as group A). Each 100 mL of PPF5% (Octapharma Pharmazeutika Produktionsgesellschaft m.b.H., Vienna, Austria) contains 5 g of selected plasma proteins buffered with sodium carbonate and stabilized with 0.004 M sodium caprylate and 0.004 M acetyl tryptophan. The plasma proteins consist of approximately 88% normal human albumin, 12% alpha and beta globulins, and no more than 1% gamma globulin as determined by electrophoresis [10]. Twenty-three patients were analyzed after the exclusion of two patients due to missing data.

Twenty-three patients received an intraoperative intravenous infusion of crystalloid solution (lactated Ringer's solution) (designated as group R). Among them, 21 patients were included in the analysis, with two patients excluded due to missing data. The contents of Ringer's lactate comprise sodium, chloride, potassium, calcium, and lactate in the form of sodium lactate, mixed into a solution with an osmolarity of 273 mOsm/L and a pH of approximately 6.5 [10].

Fluid administration was aimed to achieve a euvolemic state, defined as a central venous pressure (CVP) of 5-10 mmHg or a stroke volume variation (SVV) of ≤ 13%. Moreover, during liver resection, a CVP of -1 to 1 mmHg or an SVV of 18-21% was targeted.

General anesthesia was initiated utilizing propofol 2% and fentanyl. Subsequently, all patients were intubated and artificially ventilated to maintain arterial partial pressure of carbon dioxide (pCO2) within the standard range of 40 mmHg. An ultrasound-guided central venous catheter was inserted into the right internal jugular vein following the induction of anesthesia. Additionally, an arterial catheter, sized 20 gauge, was inserted into the radial artery.

All patients were administered 20-40 mg of furosemide to ensure adequate diuresis and CVP control. Additionally, a 30-45-degree head-up position was maintained throughout the procedure.

Arterial blood samples were collected via the radial artery catheter at various time intervals, including before the commencement of surgery, at the initiation of liver parenchymal transection (T0), at the onset of liver resection (TR), at post-liver resection (TAR), and at the conclusion of the procedure (TE).

Arterial blood gas parameters, encompassing pH, Na+, K+, Cl-, HCO3-, base deficit, and lactate concentrations, were assessed at each designated time point (T0, TR, TAR, and TE). Furthermore, hemoglobin and hematocrit concentrations, as well as albumin levels and clotting index using a rotation thromboelastometry (ROTEM) machine (Werfen GmbH, Munich, Germany), were measured and documented at time intervals T0, TR, TAR, and TE.

IAM in grams was calculated based on PV, which is calculated according to Nadler's formula for calculating total blood volume (TBV) in adults [11]: $\text{PV}=\text{TBV}&times;\left(1-\text{hematocrit}\right)$. TBV for males and females were $\left(0.006012&times;\text{H3}\right)+\left(14.6&times;\text{W}\right)+604$ and $\left(0.005835&times;\text{H3}\right)+\left(15&times;\text{W}\right)+183$, respectively. Hence, $\text{IAM}=\text{PV}&times;\left(P-alb\right)$ where H is the height in inches, which is then cubed, W in pounds is the body weight in kilograms, IAM is the intravascular albumin mass, and P-alb is the plasma albumin.

The following link is used for the online calculation of PV: http://apheresisnurses.org/apheresis-calculators/.

Systolic and diastolic blood pressure, mean arterial blood pressure, and CVP values were recorded at times T0, TR, TAR, and TE.

Tissue perfusion and blood flow were indirectly assessed through the measurement of transcutaneous oxygen tension (PtcO2) and PtcCO2, with the PtcCO2/FiO2 ratio calculated accordingly. This was accomplished by anteriorly applying a heated transcutaneous sensor (Novemetrics, Wallingford, Connecticut, United States) from the radiometer on either the left or right shoulder following a two-point calibration process. To prevent skin irritation, the sensor was repositioned every 6-8 hours, allowing 10-20 minutes for equilibration. Measurements were continuously displayed and recorded at five-minute intervals. Analyzed values were obtained at times T0, TR, TAR, and TE. Furthermore, the clotting index was measured at these four time points using ROTEM.

**Figure 1:**
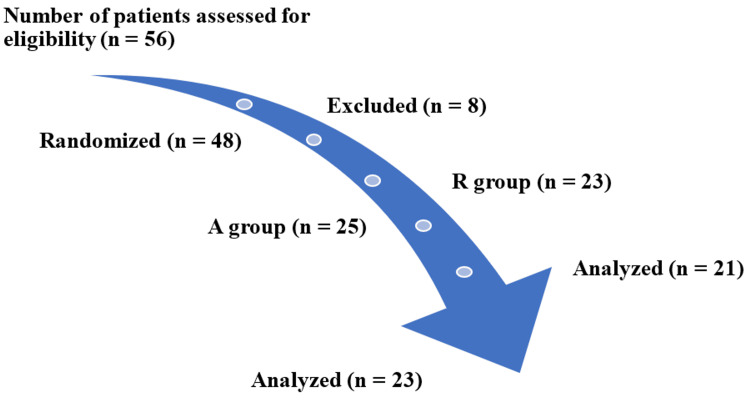
Study flow diagram n: sample size

The primary endpoint of the study focused on evaluating changes in blood acid-base status and electrolytes, employing Stewart's quantitative acid-base chemistry approach [12,13] $\text{Strong ion difference (SID)}=\text{Sodium (Na+)}-\text{Chloride (Cl-)}-\text{(Lactate)}$ where SID represents the difference between the sum of concentrations of all strong cations and the sum of all strong anions (K, Mg, and Ca are excluded). Figure 1 illustrates the study flow diagram.

The secondary endpoints are changes in perfusion markers' urine output, lactic acid, and calculated PtcCO2/FiO2 ratio and perfusion index. Hemoglobin and hematocrit concentrations, IAM, changes in PV, blood loss, and clotting index.

Statistical analyses

Statistical analyses were conducted to compare the impact of using PPF5% against crystalloids on the alteration in base deficit. With a study power of 80%, an expected change in the standard deviation (SD) of 0.45, and a significance level of p<0.05, the required sample size was determined to be 44 patients (22 in each group). To account for a potential 10% dropout rate, 24 patients per arm were deemed sufficient, providing reasonable confidence to ascertain whether a fundamental distinction exists between both groups [14].

The data was presented as mean±SD or percentages. Comparisons between variables in groups A and R were conducted using unpaired two-tailed Student's t-tests. For unpaired parametric data, including the volume of crystalloid infused and urine output, Mann-Whitney U tests were employed. Statistical analyses were carried out utilizing IBM SPSS Statistics for Windows, Version 23.0 (Released 2015; IBM Corp., Armonk, New York, United States), with a significance level set at p<0.05.

General linear modeling (GLM) was employed for multivariate repeated measurement analysis to elucidate the effects (within subjects and between subjects) of repeated time measures. The GLM repeated measures procedure was based on the general linear model, assuming linear relationships between factors and covariates with the dependent variables.

The procedure generated a model encompassing all factorial interactions, implying that each combination of factor levels could exert a distinct linear effect on the dependent variable. Profile plots were utilized to display the model-estimated means for the two groups at each time point captured in the study, with time points plotted on the horizontal axis and separate lines representing each group.

## Results

The investigation adhered to the study protocol by tracking patient attributes alongside fluctuations in hemoglobin, hematocrit, PPF5%, Ringer's lactate, and urine output.

The time point analysis revealed statistically significant disparities in the mean values of PPF5% between the groups at time points TAR and TE, as indicated in Table 1. Conversely, no notable variations were observed in SVV and CVP variables when comparing group A to group R across distinct measurement intervals.

**Table 1:** Patients' characteristics and changes in hemoglobin, hematocrit, PPF5%, Ringer's lactate, and urine output BMI: body mass index; n: number; M: mean; MD: median; SD: standard deviation; PPF5%: human plasma protein fraction 5% *p≤0.05 is considered statistically significant at a 99% confidence level. T0 represents the time at the start of surgery, TE represents the time at the end of surgery, and TAR represents the time after liver resection. *p≤0.001 was considered statistically significant at a 99% confidence level. ^†^Data proportions tested using Pearson's chi-squared test. ^‡^Normality data distribution; statistical testing done using Student's t-test; represented as M±SD. ^§^Non-normality data distribution; statistical testing done using Mann-Whitney U test of independence; represented as MD (IQR).

	Group R (n=21)	Group A (n=23)	*P-value
Female (^†^gender)	8 (38.1%)	8 (32%)	0.760
Male (^†^gender)	13 (61.9%)	17 (68%)	
^‡^Age (years)	45.8±13.2	51.5±12.4	0.147
^§^BMI (kg/m^2^)	26 (6.67)	25 (7.23)	0.419
^§^Total Ringer's lactate (ml)	2412 (972)	744± (947)	<0.001*
^§^Total PPF5%	325 (707)	1795 (528)	<0.001*
IAM (g) at TAR	102.2 (31.1)	125.7±34.4	0.02*
IAM (g) at TE	96.6 (29.4)	139.5±34.3	<0.001*
^‡^Hemoglobin at T0	13.6±1.7	12.7±2.4	0.135
^‡^Hemoglobin at TE	12.2±1.8	9.9±1.8	0.001
^‡^Hematocrit at T0	41.8±5.4	38.9±7.6	0.137
^‡^Hematocrit at TE	31.2±5.8	31±6.1	0.001
^§^Urine output at T0 (ml)	100 (220)	100 (260)	0.830
^§^Urine output at TE (ml)	350 (300)	263.3 (125)	0.112

The interaction effect within subjects' IAM (gm/l) demonstrated statistical significance, revealing a linear interaction effect (p<0.05) in the final modeling, as evidenced by notable alterations in variables at times TAR and TE, with significant changes observed (125.75 vs 103.51 and 139.57 vs 97.11; p<0.05). Individual time point analysis further underscored the significant disparity in mean PPF5% values between the groups at time points TAR and TE, as delineated in Table 1. Conversely, no significant fluctuations were observed in SSV and CVP variables when comparing group A to group R across various measurement times.

Changes in acid-base and electrolytes (SID)

**Figure 2:**
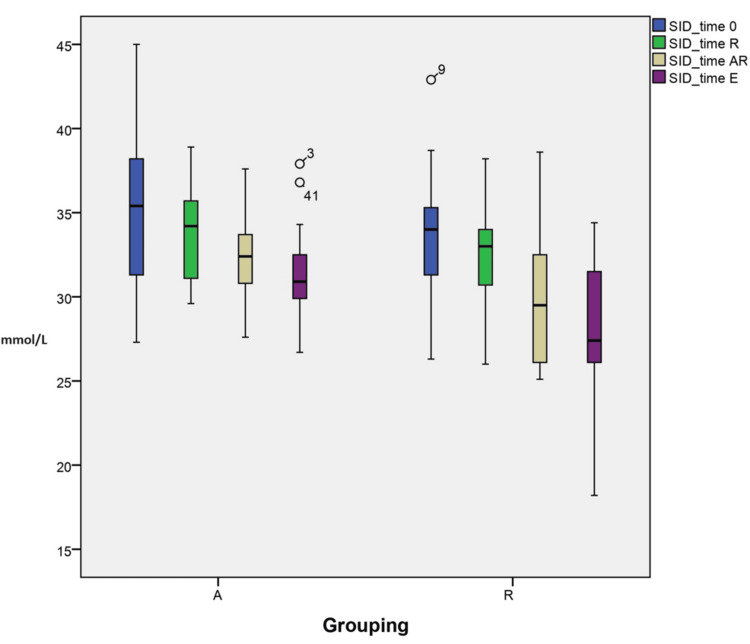
Changes in acid-base and electrolytes (strong ion difference)

The variations in SID at different time points are explained by grouping (A vs R) interactions. These within-subject interactions were statistically significant to the final modeling, as suggested in the profile plots (p<0.001) (Figure 2). Furthermore, individual time point analysis showed that the difference in means and SID between the groups is statistically significant (p=0.019 and p=0.008, respectively) at TAR and TE, respectively (32.2±2.59 vs 30.05±4.17 and 31.3±2.56 vs 28.2±3.9, respectively). 

Changes in acid-base and electrolytes (sodium)

**Figure 3:**
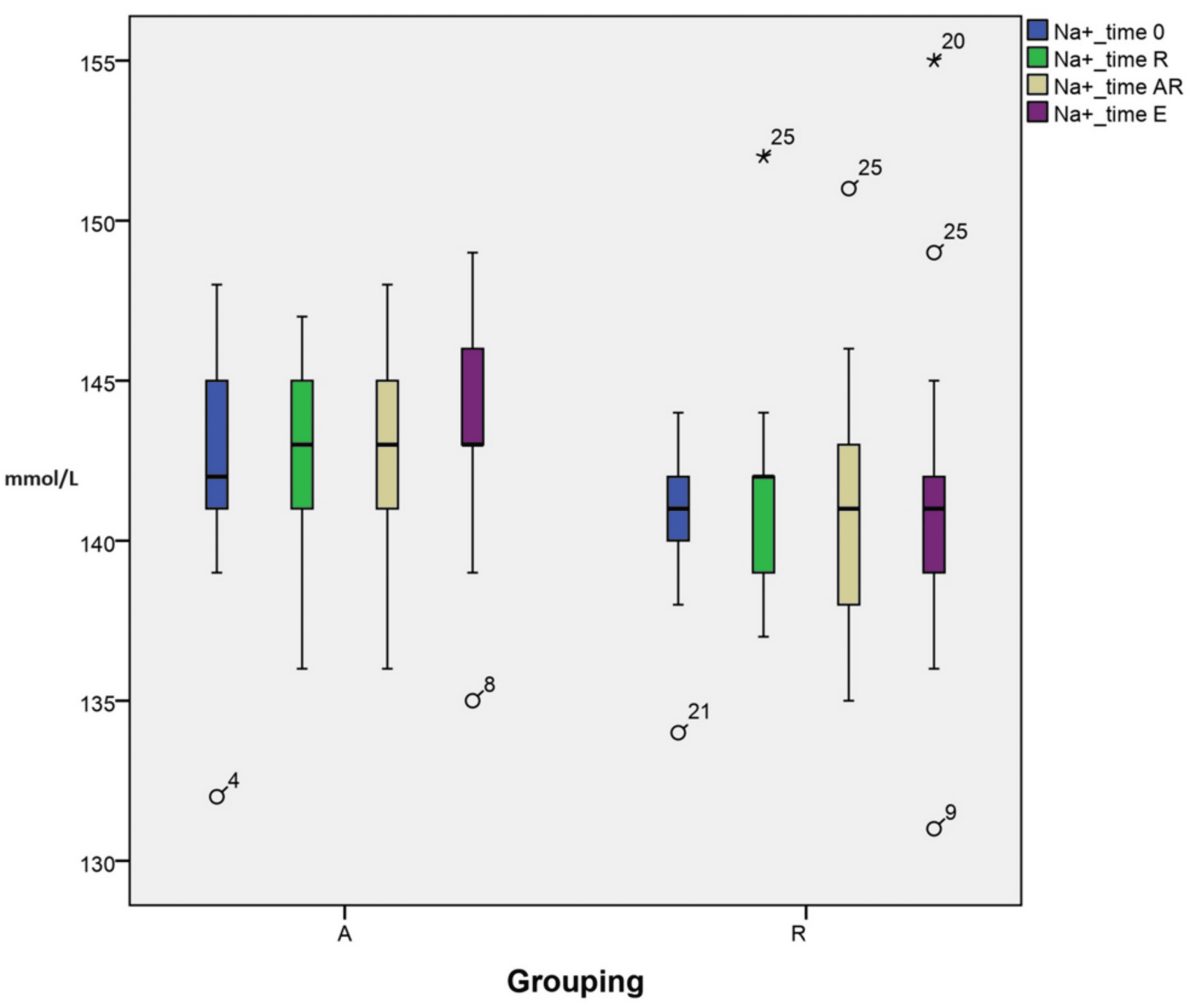
Changes in acid-base and sodium

The variations in sodium at different time points are explained by grouping (A vs R) interactions. These within-subject interactions were statistically significant (p=0.027) to the final modeling, as suggested in the profile plots (Figure 3). Furthermore, individual time point analysis showed that the difference in means and SID (142.6±2.77 vs 140.9±3.65 TAR and 143.8±2.9 vs 141.0±4.8 TE) between the groups is statistically significant at TAR and TE, respectively. 

Changes in acid-base and electrolytes (lactate)

**Figure 4:**
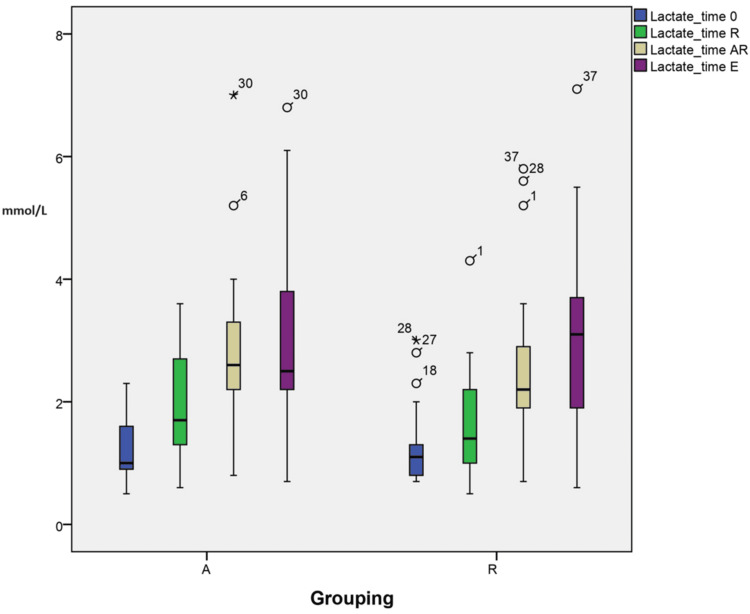
Changes in acid-base and lactate

The plot for lactate shows that group A has slightly better readings with less lactate after resection and at the end of surgery (2.84±1.26 vs 2.5±1.4 TAR and 3.0±1.4 vs 3.07±1.5 TE). However, this change was not statistically significant when groups were compared together (p=0.218). There is a significant change between variables at different time points within each group (p<0.05), as illustrated in Figure 4.

Changes in acid-base and electrolytes (chloride)

The plot for chloride shows that group A has better readings with less chloride after resection and at the end of surgery. However, chloride in group A was statistically insignificant compared to group R at the end of surgery.

Changes in bicarbonate

**Figure 5:**
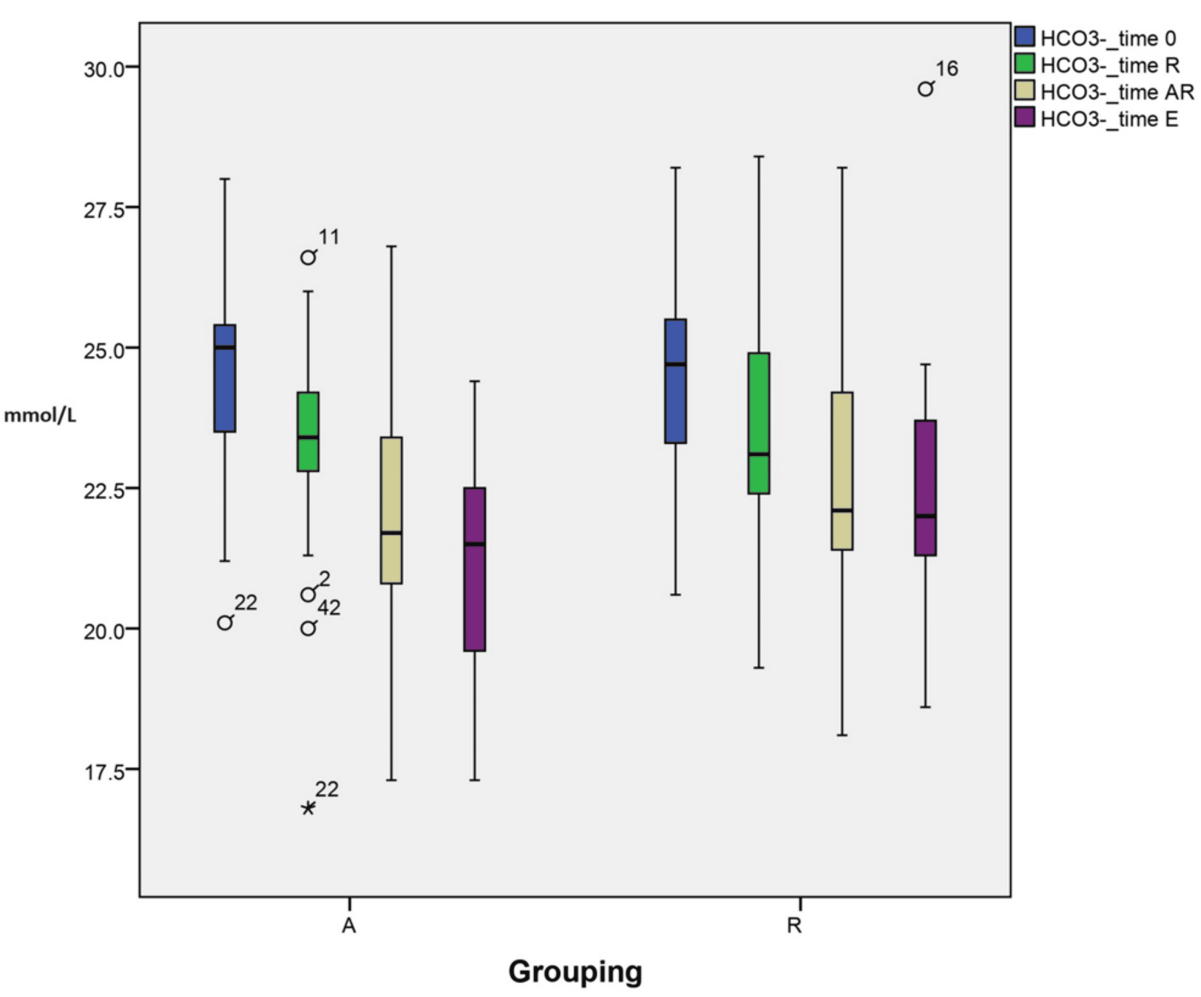
Changes in bicarbonate

The variations in bicarbonate at different time points are explained by grouping (A vs R) interactions. These within-subject interactions were statistically significant to the final modeling, as suggested in the profile plots (p<0.001). In addition, individual time point analysis showed that the difference in means and SD between the groups is statistically significant (p=0.049) lower in group A compared to group R at the end of surgery (21.0±2.16 vs 22.37±2.36 TE), as illustrated in Figure 5.

Changes in pH and base deficit

**Figure 6:**
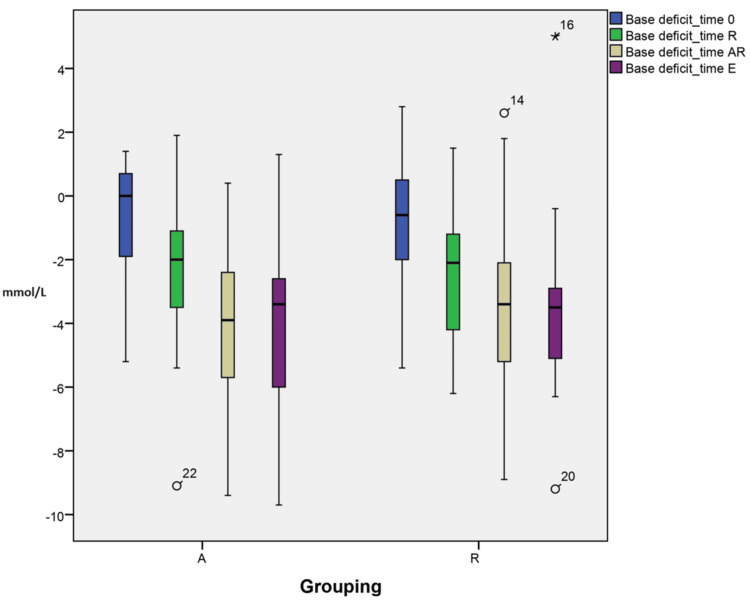
Changes in base deficit

**Figure 7:**
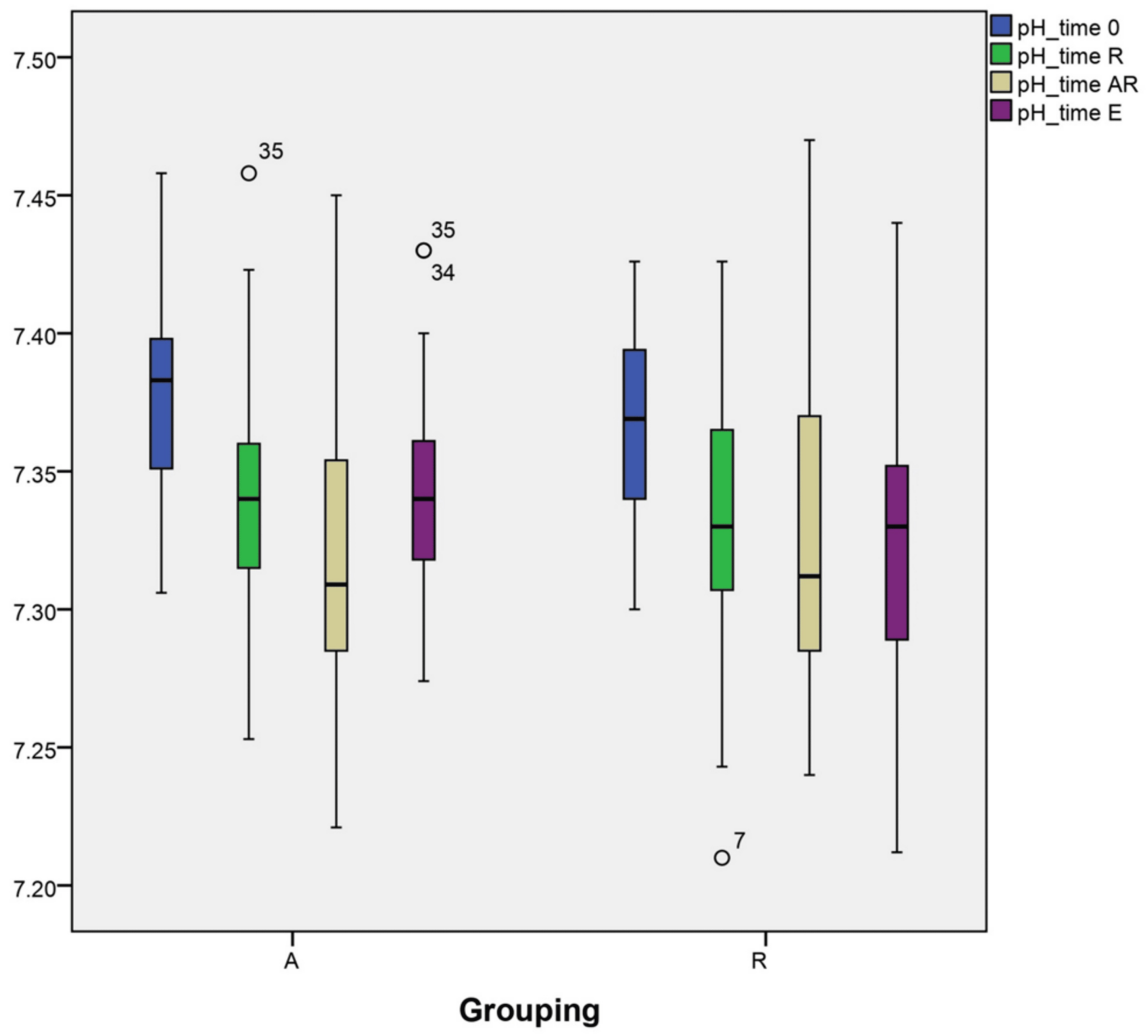
Changes in pH

Despite the lower base deficit in group A, the variations in base deficit and pH at different time points could not be explained by grouping (A vs R) interactions. There are no significant changes in pH and base deficit when the two groups, A and R, are compared at the end of surgery (-4.076±2.68 vs -3.48±2.71, respectively) (7.31±0.04 vs 7.32±0.05 TAR and 7.34±0.04 vs 7.32±0.049 TE). These within-subject interactions were statistically significant (p=0.003) to the final modeling, as suggested in the profile plots, as illustrated in Figure 6 and Figure 7.

Changes in perfusion markers

Tissue perfusion was measured by PtcCO2/FiO2 ratio which was measured at each time point of the study (T0: time baseline measurement after induction of anesthesia, TR: time at liver resection, TAR: time after resection, TE: time at end of surgery) and showed that there were no differences in means and standard deviations between the study groups except for TAR and TE (Table 2).

**Table 2:** Tissue perfusion (PtcCO2/FiO2 ratio) Tissue oxygen/FiO2 at the time after resection (TAR) and at the time at end of surgery (E). TE represents the time at the end of surgery, and TAR represents the time after liver resection. *p≤0.05 is considered statistically significant at a 99% confidence level.

	PtcCO2/FiO2 ratio±SD		P-value
	Group R	Group A	
TAR	200±119	273±77	0.032*
TE	231±145	303±76	0.049*

Changes in hemoglobin, hematocrit, blood loss, and clotting index

There was no significant correlation between the two groups to blood loss and clotting index at TAR and TE (correlation coefficient: -0.1415 and -0.219, respectively) (p=0.403 and p=0.186, respectively). However, there is significantly low hemoglobin and hematocrit in group A compared to group R at the end of surgery (TE) as shown in Table 1.

## Discussion

In this randomized controlled trial, our hypothesis posits that employing PPF5% instead of Ringer's lactate in patients undergoing liver resection surgery will enhance tissue perfusion and the blood's buffering capacity. Liver resection surgery poses several challenges, including fluctuations in intravascular volume due to dehydration during the procedure and subsequent hydration post-resection.

Manipulation of the liver during liver resection surgery induces liver cell injury, triggering an inflammatory response that results in elevated liver markers, independent of intermittent hepatic inflow occlusion or anesthetic effects [15]. Moreover, alterations in blood chemistry may manifest preoperatively, particularly in patients with chronic liver disease, as the liver is the site of albumin synthesis. Chronic liver disease often leads to albumin deficiency, which serves as a crucial determinant of plasma osmotic pressure and performs various physiological roles, including acting as a carrier for several compounds, exerting scavenger, antioxidant, and anti-inflammatory effects, and contributing to pH homeostasis as a buffer molecule [7-16]. Notably, Figge et al. delineated albumin's acid-base characteristics in human blood, while Stewart established the relationship between albumin dissociation and pH through six formulas, concluding that the addition of a weak acid to a CO2 solution diminishes the buffering capacity of the solution [17]. In summary, the buffering strengths of albumin solutions, under standard electrolyte composition and pCO2 levels, are inversely proportional to its concentration.

Cohen and Woods categorized the causes of lactic acidosis into two main types: type A and type B1. Type A is associated with inadequate tissue oxygen delivery, whereas type B1 is linked to liver failure, notwithstanding impaired tissue oxygenation, given the liver's significant role in lactate clearance, which accounts for up to 50-70% of whole-body lactate clearance. Lactic acidosis induces detrimental effects such as decreased cardiac output, vasodilation, cardiac arrhythmias, and even sudden death [18].

The elevation in lactate levels during liver resection surgery can be attributed to various factors including the effect of anesthesia, preexisting liver disease, temporary vascular occlusion during resection, liver tissue hypoxia resulting from manipulation, and the subsequent increase in the conversion of pyruvate to lactic acid. This process is exacerbated by vasopressor administration, endogenous stress hormones, and stored blood administration [19,20]. In our study, a significant increase in tissue perfusion was observed in group A compared to group R, alongside a significant increase in lactate levels in both groups, with no significant change in lactic acid when comparing the two groups. These findings suggest that lactic acid production during liver surgery is not influenced by the type of fluid used or overall tissue perfusion but rather by local factors related to the surgery.

Contrary to our study, Shin et al. demonstrated in their randomized controlled study that non-lactate-containing crystalloid solutions may offer significant advantages over Ringer's lactate solution concerning lactate and liver profiles in living donors undergoing right hepatectomy. These benefits include lower serum lactate, pH, bilirubin, and prothrombin time, with no difference in mortality and hospital stay [8].

Utilizing the Stewart and Fencl model to elucidate acid-base changes associated with PPF5%, we discovered that the primary change in SID in the albumin-rich PPF5% group is linked to high sodium, whereas in the Ringer's lactate group, it is associated with changes in chloride. This implies that PPF5% induces hypernatremia alkalosis, while Ringer's lactate induces hyperchloremic acidosis. However, an increase in blood chloride in group A at TE remains unexplained, unless PPF5% contains chloride or it is secondary to the Ringer's lactate used in combination with PPF5% in this group [10].

Furthermore, according to Figge's modification, there is an increase in effective SID (SIDe) compensating for the change in SID, considering the addition of weak acids like albumin to the blood, provided that other variables remain stable, including the increase in lactic acid (endogenous) or sodium (albumin solvent) observed in this study [7-21].

The expected increase in SIDe limits the change in strong ion gap (SIG) (SIG=SID-SIDe) in the albumin group. Consequently, a normal SIG indicates minimal or no change in electrolytes, pH, and acid-base balance in patients receiving albumin-rich PPF5% compared to crystalloids. Overall, both fluids exhibit a similar effect on pH and SBE.

Finally, the study did not detect a significant effect of large-volume transfusion of albumin-rich PPF5% compared to Ringer's solution on blood loss and coagulation, contrary to previous findings suggesting that albumin transfusion reduces clot strength. The lower hematocrit in the albumin group may be attributed to the hemodilution effect of PPF5% [22].

Our study exhibits several limitations. Firstly, the sample size is relatively small, potentially limiting the generalizability of our findings. Secondly, while the study design is randomized, it lacks blinding, introducing a potential source of bias. Thirdly, critically ill patients were not included in the study, which may impact the extrapolation of our results to this population. Lastly, cardiac patients were excluded from the study, as their hypoperfusion status could potentially influence acid-base balance parameters.

## Conclusions

The utilization of PPF5% in substantial volumes for the rehydration of patients undergoing liver resection surgery appears to be safe, as evidenced by the absence of significant alterations in acid-base balance, electrolyte levels, and coagulation parameters when compared to the administration of Ringer's lactate solution. Moreover, the use of PPF5% demonstrates enhanced tissue perfusion and increased hemodilution, further supporting its efficacy in this clinical setting.
